# Microglial dynamics after axotomy-induced retinal ganglion cell death

**DOI:** 10.1186/s12974-017-0982-7

**Published:** 2017-11-09

**Authors:** Francisco M. Nadal-Nicolás, Manuel Jiménez-López, Manuel Salinas-Navarro, Paloma Sobrado-Calvo, Manuel Vidal-Sanz, Marta Agudo-Barriuso

**Affiliations:** 1 Grupo de Oftalmología Experimental, Instituto Murciano de Investigación Biosanitaria-Virgen de la Arrixaca, Edificio LAIB Planta 5ª, Carretera Buenavista s/n, 30120 El Palmar, Murcia Spain; 2 Departamento de Oftalmología, Facultad de Medicina, Universidad de Murcia, Murcia, Spain; 3 Present address: Retinal Neurophysiology Section, National Eye Institute, National Institutes of Health, Bethesda, MD 20892 USA

**Keywords:** Phagocyte, Iba1, Brn3a, Transcellular labeling, Migration, Fluorogold

## Abstract

**Background:**

Microglial cells (MCs) are the sentries of the central nervous system. In health, they are known as surveying MCs because they examine the tissue to maintain the homeostasis. In disease, they activate and, among other functions, become phagocytic to clean the cellular debris. In this work, we have studied the behavior of rat retinal MCs in two models of unilateral complete intraorbital optic nerve axotomy which elicit a different time course of retinal ganglion cell (RGC) loss.

**Methods:**

Albino Sprague-Dawley rats were divided into these groups: (a) intact (no surgery), (b) fluorogold (FG) tracing from the superior colliculi, and (c) FG tracing + crush or transection of the left optic nerve. The retinas were dissected from 2 days to 2 months after the lesions (*n* = 4–12 group/lesion and time point) and then were subjected to Brn3a and Iba1 double immunodetection. In each intact retina, the total number of Brn3a^+^RGCs and Iba^+^MCs was quantified. In each traced retina (b and c groups), FG-traced RGCs and phagocytic microglial cells (PMCs, FG^+^Iba^+^) were also quantified. Topographical distribution was assessed by neighbor maps.

**Results:**

In intact retinas, surveying MCs are homogenously distributed in the ganglion cell layer and the inner plexiform layer. Independently of the axotomy model, RGC death occurs in two phases, one quick and one protracted, and there is a lineal and topographical correlation between the appearance of PMCs and the loss of traced RGCs. Furthermore, the clearance of FG^+^RGCs by PMCs occurs 3 days after the actual loss of Brn3a expression that marks RGC death. In addition, almost 50% of MCs from the inner plexiform layer migrate to the ganglion cell layer during the quick phase of RGC loss, returning to the inner plexiform layer during the slow degeneration phase. Finally, in contrast to what happens in mice, in rats, there is no microglial phagocytosis in the contralateral uninjured retina.

**Conclusions:**

Axotomy-induced RGC death occurs earlier than RGC clearance and there is an inverse correlation between RGC loss and PMC appearance, both numerically and topographically, suggesting that phagocytosis occurs as a direct response to RGC death rather than to axonal damage.

## Background

In rodents, retinal ganglion cell (RGC) loss after optic nerve axotomy is a well-documented process that occurs in two phases [1]. The first one is quick and devastating, lasts around 14 days, and causes the loss of ~ 85% of RGCs. Then, RGC death takes a slower but steady pace in such a way that 15 months after the injury, around 1% of the original population remains [2]. Furthermore, in rats, the rate of RGC loss is modulated by the distance from the optic nerve head at which the axotomy is performed, slower with longer distances [2, 3].

After axotomy, damaged RGCs die by apoptosis [4, 5]. Apoptotic cells initiate a highly controlled cascade of events that finishes when the cell bodies are cleared from the tissue by phagocytes [6, 7] which, in the central nervous system, are the microglial cells (MCs).

In the healthy retina, MCs are located on four layers: the retinal nerve fiber layer (RNFL) that contains RGC axons; the ganglion cell layer (GCL), where RGC somatas and displaced amacrine cells lie; the inner plexiform layer (IPL); and the outer plexiform layer (OPL). While plexiform layers are mainly formed by neuropil, the IPL also contains displaced RGCs (dRGCs) [8].

MC surveys the tissue (surveying MCs (SMCs)), keep the homeostasis, and stabilize the synapses [9, 10]. Upon neuronal death, MCs activate [11, 12] and migrate to the damaged region [13] to participate in the careful clearance of cellular debris and to repair the local damage (reviewed in [14–16]). This removal is critical to maintain tissue integrity (reviewed in [17]).

The phagocytic uptake of MCs can be measured in vitro by different assays such as administration of fluorescent bioparticles or cell debris previously labeled [18]. In vivo, phagocytic microglial cells (PMCs) can be identified by transcellular labeling, a process that occurs when a MC engulfs a dying fluorescent neuron [19–24]. This is a powerful approach to study PMC behavior and tissue clearing, provided that a given neuronal population is first labeled and then injured.

In rats and mice, almost the whole population of RGCs (~ 98% [25, 26]) can be traced from both superior colliculi with fluorogold (FG), a fluorescent retrograde tracer. Thus, if after RGC tracing the optic nerve is injured, it is possible to asses at the same time RGC clearance and the appearance of transcellularly labeled MCs [21–24]. Using this tool in mice, we have shown that after unilateral axotomy, the appearance of PMCs and the clearance of traced RGCs in the injured retina are linearly correlated [21, 24]. Interestingly, in the contralateral uninjured retina of pigmented mice, some MCs become phagocytic and their number and distribution are constant and independent of RGC survival in the injured retina.

There are many works describing the microglial response to injury, activation mechanisms and phenotypes, and release of inflammatory mediators or their involvement in neurodegeneration and neuroprotection [27–33]. But little is known of their numerical and topographical dynamics and their correlation with the dying neurons, for instance, is the temporal and topographical course of PMC appearance regulated by the course of neuronal death? Is neuronal clearance the same as neuronal death? Is there a contralateral retinal response involving PMC appearance in adult rats? Do MCs travel from the outer retinal layers to the inner retina to cope with the massive RGC death triggered by axotomy?

To answer these questions, we have used RGC tracing and immunodetection of microglial cells (Iba1) and viable RGCs (Brn3a) in the same retinas after two different axotomy models.

## Methods

### Animal handling

Adult female albino Sprague-Dawley rats (200 g, *n* = 80) were obtained from the University of Murcia breeding colony. All animals were treated in compliance with the European Union guidelines for Animal Care and Use for Scientific Purpose (Directive 2010/63/EU) and the guidelines from the Association for Research in Vision and Ophthalmology (ARVO) Statement for the Use of Animals in Ophthalmic and Vision Research. All procedures were approved by the Ethical and Animal Studies Committee of the University of Murcia, Spain (REGA 300305440012).

Animals undergoing surgery were anesthetized by intraperitoneal injection of a mixture of ketamine (60 mg/kg; Ketolar, Pfizer, Alcobendas, Madrid, Spain) and xylazine (10 mg/kg; Rompum, Bayer, Kiel, Germany). Analgesia was provided by subcutaneous administration of buprenorphine (0.1 mg/kg; Buprex, buprenorphine 0.3 mg/mL; Schering-Plough, Madrid, Spain). During and after surgery, the eyes were covered with an ointment (Tobrex; Alcon, S. A., Barcelona, Spain) to prevent corneal desiccation.

The animal groups are the following: (i) intact, no surgery; (ii) tracing controls, tracing from the superior colliculi (SCi), analyzed 7 days or 2 months later (9 weeks); (iii) axotomized, tracing from the SCi, and 1 week later optic nerve crush (ONC) or optic nerve transection (ONT), retinal analysis 2, 5, 9, 14 days or 2 months (9 weeks tracing) later. Injured retinas are the left ones, and contralateral retinas the right ones.

### Retrograde tracing

After the exposure of both SCi, a pledge of gelatin sponge soaked in 3% fluorogold (FG, Fluorochrome, LLC, USA) dissolved in 10% dimethyl sulfoxide/saline was applied onto both SCi 7 days prior optic nerve injury, or 7 days or 9 weeks before sacrifice (traced but otherwise intact retinas), following standard techniques in our laboratory [8, 23, 24, 34, 35].

### Optic nerve injuries

Seven days after RGC tracing and according to previous studies, the left optic nerve was crushed or transected at 3 or 0.5 mm from the optic disc, respectively [2, 4, 34–36]. With these settings, ONC elicits a slower RGC death than ONT [2, 34].

### Tissue preparation and sectioning

Unless otherwise stated, all reagents were from Sigma-Aldrich Quimica S.A. Madrid, Spain.

Animals were sacrificed with an intraperitoneal injection of an overdose of sodium pentobarbital (Dolethal, Vetoquinol; Especialidades Veterinarias, S.A., Alcobendas, Madrid, Spain). All animals were perfused transcardially with 0.9% saline solution followed by 4% paraformaldehyde in 0.1 M phosphate buffer.

The retinas (*n* = 4–12/group and time point, see tables) were dissected as flattened whole mounts as previously described [34]. Some eye cups (*n* = 3/group and time point) were cryoprotected in a series of sucrose gradients, embedded in optimal cutting temperature medium (OCT, Tissue-Tek, Sakura-Finetek, VWR, Barcelona, Spain), frozen, and sectioned in a cryostat at 14-μm thickness.

### Immunohistofluorescence

Double immunodetection of RGCs and MCs was carried out as previously described [2, 11, 21, 35, 37]. Healthy RGCs were detected using goat α-Brn3a (C-20; Santa-Cruz Biotechnology, Heidelberg, Germany) diluted 1:750 and microglial cells using rabbit α-Iba1 antibody (ionized calcium-binding adapter molecule 1; Wako Chemicals GmbH, Neuss, Germany) diluted 1:1000. Secondary detection was carried out with donkey α-goat and donkey α-rabbit (Alexa Fluor 594 and 488 respectively; Molecular Probes; Thermo Fisher Scientific, Madrid, Spain) diluted at 1:500. In some retinal sections, all nuclei were counterstained using antifading mounting media with DAPI (4′,6-diamidino-2-phenylindole, Vectashield mounting medium with DAPI; Vector Laboratories, Palex Medical, Barcelona, Spain).

### Image acquisition

Whole-mounted retinas where photographed focusing firstly on the GCL and secondly on the IPL. All images were acquired using an epifluorescence microscope (Axioscop 2 Plus; Zeiss Mikroskopie, Jena, Germany) equipped with a computer-driven motorized stage (ProScan H128 Series; Prior Scientific Instruments, Cambridge, UK) controlled by image analysis software (Image-Pro Plus, IPP 5.1 for Windows; Media Cybernetics, Silver Spring, MD). Retinal photomontages were reconstructed from 154 (11 × 14) individual images by zigzag tiling, as reported [2, 25, 38].

### Quantification

FG^+^RGCs in intact and contralateral to the lesion retinas, and Brn3a^+^RGCs in all retinas, were automatically quantified using our previously described methods [25, 34]. After injury, microglial cells became transcellularly labeled (PMCs: FG^+^Iba1^+^), being for the automated routine impossible to tell them apart from traced RGCs Thus, FG^+^RGCs were manually quantified (FG^+^Iba1^−^) and their total number estimated using previously reported methods [11]. Briefly, the retinas were divided into three concentric rings (central, equatorial, and peripheral) and four samples within each were selected (0.25 mm^2^/sample). Each sample corresponded to a retinal quadrant, therefore, three samples per quadrant and four samples per ring. The area of each ring was measured, and the total number of FG^+^RGCs in a given ring were estimated from the mean number quantified in its four samples, thus giving back the number of FG^+^RGCs in the central, medial, and peripheral retina. The total number of FG^+^RGCs per retina was the sum of the partial values. This method was validated in intact retinas, where FG^+^RGCs were quantified automatically and manually (for more details, see [11]).

All non-phagocytic MCs (Iba1^+^FG^−^) and PMCs (Iba1^+^FG^+^) were manually dotted on the retinal photomontages using Adobe Photoshop CS 8.0.1; (Adobe Systems, Inc., San Jose, CA), and the dots were automatically quantified (IPP software) [2, 8, 24, 39]. Quantitative data from all populations were exported to a spreadsheet application (Microsoft Office Excel 2003; Microsoft Corporation, Redmond, WA) for further analyses.

### Spatial distribution

**Fig. 1:**
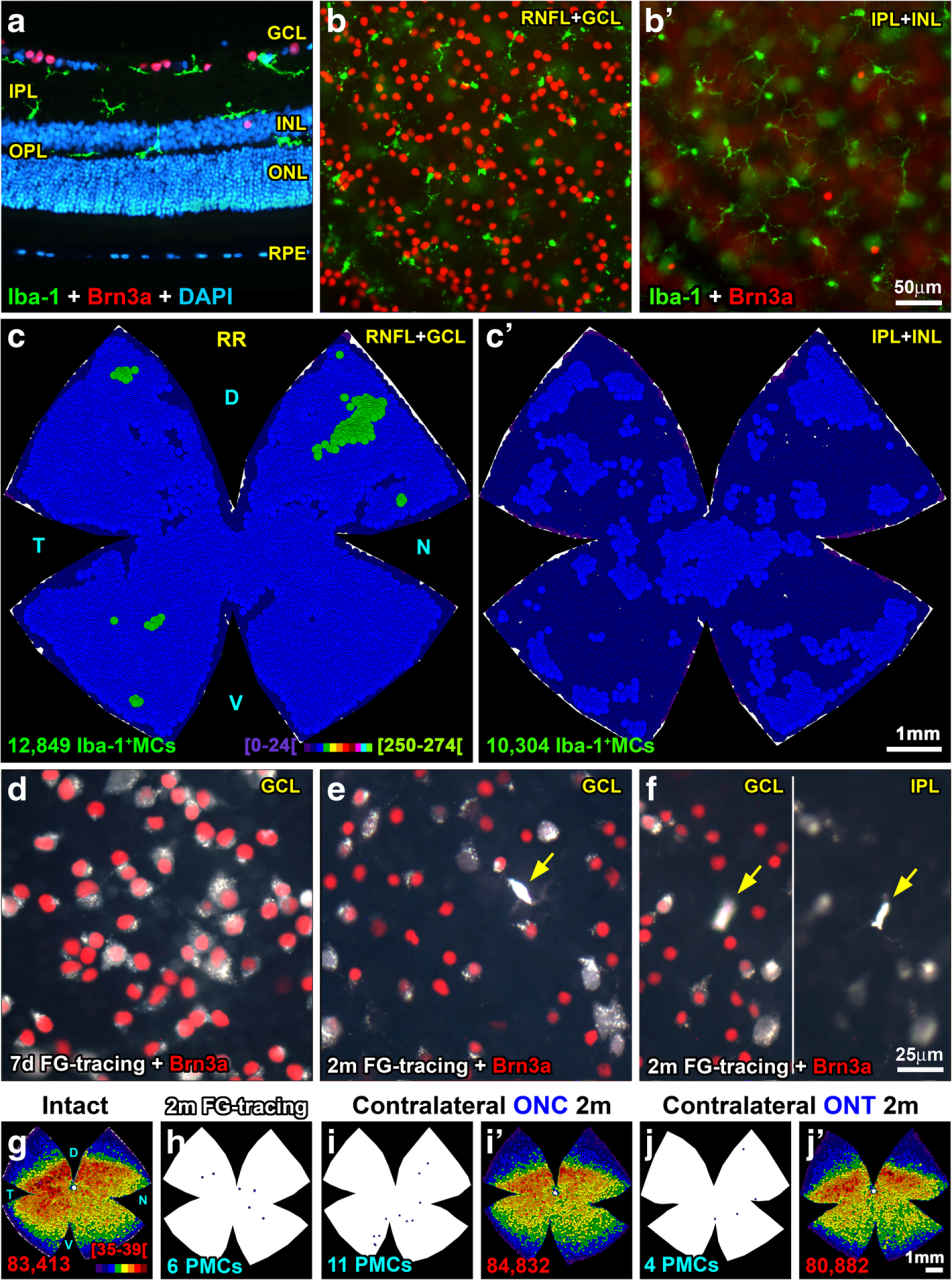
Microglial cells are homogeneously distributed in intact retinas. **a** Cross section showing the location of Iba1^+^MCs (green) and Brn3a^+^RGCs (red) in intact retinas. Note that some MCs in the IPL are lying on top of the INL. **b** Magnification from an intact flat-mounted retina focused on the RNFL/GCL showing Iba1^+^MCs and Brn3a^+^RGCs. **b’** Iba1^+^MCs and displaced Brn3a^+^RGCs in the same area as **b** but changing the focus to the IPL/INL. **c** Neighbor map of an intact retina showing the distribution of MCs in the GCL. **c’** Neighbor map from the same retina showing the distribution of MCs in the IPL. **d** Magnification from a control retina 7 days after fluorogold tracing (white), immunodetected with Brn3a (red). In these retinas, no PMCs were observed. In control retinas analyzed 2 months after tracing (**e**, **f**) some PMCs (yellow arrows) were observed in the GCL (**e**, **f** left) and IPL (**f** right). **g** Neighbor map of an intact retina showing the distribution of Brn3a^+^RGCs. **h** Retinal outline showing the position of the few PMCs found in control retinas analyzed 2 months after tracing. **i**, **j** Retinal outlines showing the location of the PMCs found in contralateral to the lesion retinas analyzed 2 months after the unilateral ONC (**i**) or ONT (**j**). **i’**, **j’** Brn3a^+^RGCs neighbor maps from the same retinas as in **i**, **j**. *For this and the subsequent figures*: The number of cells represented is shown below each map. Neighbor maps represent the number of neighbors around each cell (dot) within a given radius and color scale. For MC representation, the radius is 0.276 mm and the scale (**c**, bottom) goes from 0 to 24 (purple) to ≥ 250–274 neighbors (bright green). For RGCs, the radius is 0.0552 mm and the color scale (**g**, bottom) goes from 0 to 4 (purple) to ≥ 35–39 (dark red) neighbors. D dorsal, V ventral, T temporal, N nasal, RR right retina, RNFL retinal nerve fiber layer, GCL ganglion cell layer, IPL inner plexiform layer, INL inner nuclear layer, OPL outer plexiform layer, ONL outer nuclear layer, ONC optic nerve crush, ONT optic nerve transection, MC microglial cell, PMC phagocytic microglial cell, m months, d days

The spatial distribution of RGCs and MCs was assessed by the near neighbor algorithm as reported [2, 21, 24, 39, 40]. The position of each Brn3a^+^RGC or dot (microglial cell) in the photomontage was translated to an *X*,*Y* location using as reference the optic nerve (0,0 point). Maps were plotted using SigmaPlot (SigmaPlot 9.0 for Windows; Systat Software, Inc., Richmond, CA, USA). In addition, using a color code, these maps depict the number of neighbors around a given cell in a given radius (see Fig. 1 legend).

### Statistical analysis

Data were analyzed with GraphPad Prism v.7 (GraphPad San Diego, USA). Each cell type was analyzed independently along time within each lesion and between lesions (one-way ANOVA, post hoc Tukey’s test). Differences were considered significant when *p* < 0.05. Data are presented as mean ± standard deviation. Correlation analyses (microglial cells vs. RGCs, or cells vs. time) were modeled using the same program and were considered significant when *R* ^2^ (goodness of the fit) was ≥ 0.9.

## Results

In all the retinas except the intact ones, RGCs were identified by Brn3a immunodetection and tracing. The loss of Brn3a signal announces that a RGC is dead [2, 4]. A traced RGC might not be viable (no Brn3a expression), but it may still be detectable because the FG filling the RGC soma is an exogenous compound that will disappear from the tissue only upon microglial clearance. In intact retinas, MCs are called surveying MCs (Iba1^+^). In the experimental retinas, we counted Iba1^+^FG^+^ PMCs, and Iba1^+^FG^−^ MCs that may or may not have been activated, for accuracy, and we termed them non-phagocytic microglial cells (non-PMCs).

We could not discriminate between the RNFL and GCL. Also, we acquired images from the IPL and the inner limit of the inner nuclear layer (INL) because we used the dRGCs as pegs. For simplicity, we will refer to these layers in the text as GCL or IPL, respectively.

### Intact retinas

As observed in the retinal section in Fig. 1a, SMCs are found in the GCL, IPL, and OPL. In the IPL, some MCs lie on the inner limit of the INL. Using flat mounts, we quantified their total number in the GCL or IPL (Fig. 1b, b’), which contain, respectively, RGC axons and somata, or their dendrites and dRGCs.

The total number of SMCs in the GCL and IPL is very similar (Table 1), although slightly higher in the GCL. The ratio RGC/MC is 6:1 if we take into account only MCs in the GCL, but if we add the MCs in the IPL, that is MCs in the GC complex, this ratio becomes 3:1. In both layers, SMCs are homogenously distributed (Fig. 1c, c’, Table 1).

**Table 1:** Intact, tracing control, and contralateral to the injury retinas

		Intact (*n* = 4)	Tracing control		Contralateral (*n* = 80/72*)
			7 days (*n* = 8)	2 months (*n* = 4)	
RNFL/GCL	Brn3a^+^RGCs	82,530 ± 766	81,504 ± 798	81,368 ± 491	81,486 ± 2272
	FG^+^RGCs		81,829 ± 1246	58,948^§^ ± 3381	81,513 ± 1425
RNFL/GCL	SMCs	14,151 ± 1186			
	PMCs		0 ± 0	6 ± 2	1 ± 3
IPL/INL	SMCs	11,006 ± 989			
	PMCs		0 ± 0	1 ± 1	
GCC	SMCs	25,156 ± 1367	0 ± 0		
	PMCs		0 ± 0	7 ± 3	

Mean number ± standard deviation of Brn3a^+^RGCs, FG^+^RGCs, surveying microglial cells (SMCs), and phagocytic microglial cells (PMCs) counted in the same retinas within each group. PMCs were quantified in the retinas that had been traced with fluorogold 7 days or 2 months before sacrifice to match the tracing standard (7 days) and the longer tracing times used in the injured retinas (see Table 2)

*FG* fluorogold, *RGCs* retinal ganglion cells, *RNFL/GCL* retinal nerve fiber layer/ganglion cell layer, *IPL/INL* inner plexiform layer + inner limit inner nuclear layer, *GCC* ganglion cell complex (RFNL/GCL + IPL/INL)

^§^The number of FG^+^RGCs 2 months after tracing is significantly smaller than at 7 days because FG is not a permanent dye [39, 41, 42], while the number of Brn3a^+^RGCs remains unaltered. In 2-month traced retinas, there are some PMCs, although this is not significant compared to 7 days of tracing or intact retinas (one-way ANOVA, Tukey’s post hoc test, *p* > 0.05)

*The number of contralateral retinas amounts to 80 for Brn3a^+^RGCs and PMCs (see Table 2) and to 72 for FG^+^RGCs (2-month time point excluded). No significant differences were observed between contralateral and control or intact retinas (one-way ANOVA test, Tukey’s post hoc test, *p* > 0.05)

### Tracing controls and contralateral retinas

In traced retinas, FG is observed filling the RGC cytoplasm, while Brn3a expression is nuclear (Fig. 1e). When the tracer is left for 7 days (standard procedure), there are usually no visible PMCs (Table 1). By 2 months, few PMCs are observed, both in the GCL and IPL. Note that at 2 months, the number of FG^+^RGCs decreases because FG is not a persistent label [39, 41, 42] but the number of Brn3a^+^RGCs remains unaltered (Fig. 1f–i, Table 1). In the right retinas, contralateral to the lesion, there is no RGC loss and the presence of PMCs is testimonial (Fig. 1i, j’, Table 1).

### RGC death vs. RGC clearance

**Fig. 2:**
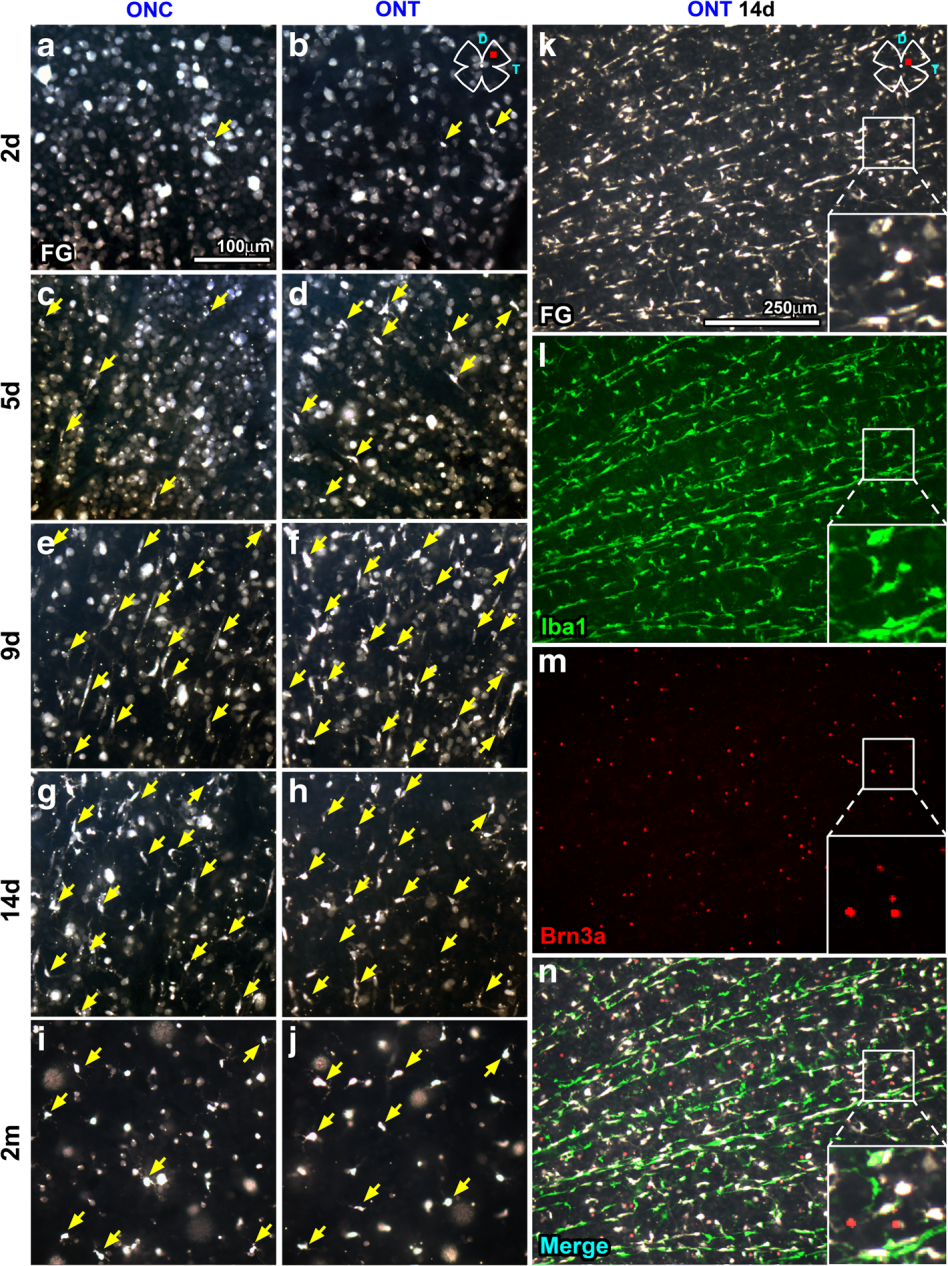
RGC clearance and PMC appearance in axotomized retinas. Magnifications from flat-mounted traced retinas analyzed at 2 (**a**, **b**), 5 (**c**, **d**), 9 (**e**, **f**), 14 days (**g**, **h**), or 2 months (**i**, **j**) after optic nerve crush (first column) or transection (second column) showing the course of FG^+^RGC disappearance and the appearance of PMCs (yellow arrows). **k**–**n** Images from the central dorso-temporal area (drawing in **k**, top right) of a representative retina analyzed 14 days after ONT and immunodetected with Iba1 and Brn3a. PMCs are those MCs transcellularly labeled (FG^**+**^Iba^**+**^). In these injured retinas, majority of FG^**−**^Iba^**+**^ MCs are probably activated, but have not yet engulfed a traced RGC

**Fig. 3:**
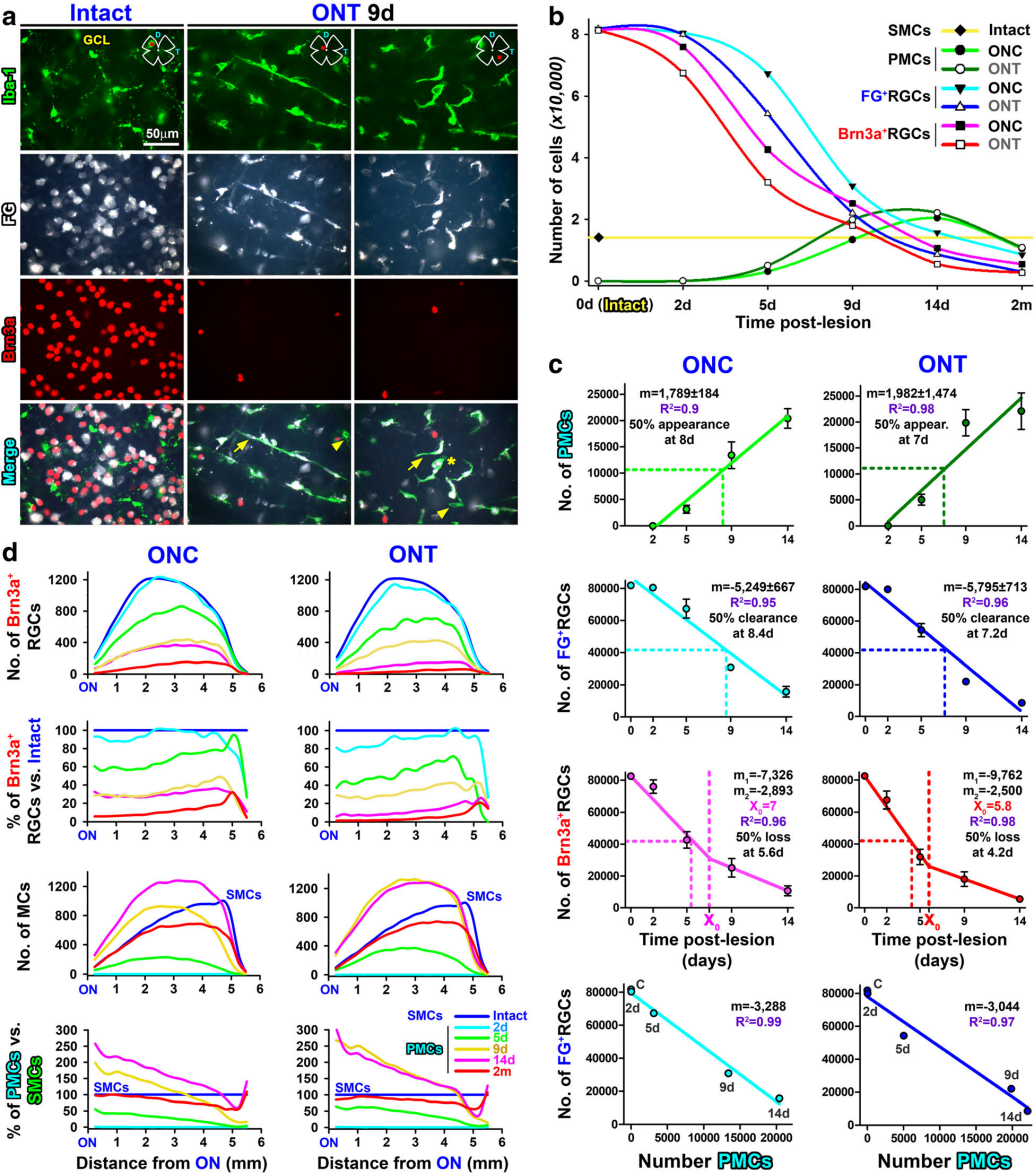
Microglial cell morphologies, quantitative topography, and correlation analyses of PMC appearance, RGC clearance, and RGC death. **a** Microglial cells in control or axotomized retinas: magnifications from control or injured FG-traced retinas immunodetected for Iba1 and Brn3a taken from the red areas shown in the retinal drawing. Arrows, PMCs. Arrowheads, activated MCs that have not yet engulfed a traced RGC. Asterisk, MC engulfing a traced RGC that has lost its Brn3a expression. **b** Temporal course of RGC loss and PMC appearance. The yellow line is the number of SMCs in intact retinas. **c** Correlation analyses created from total mean numbers ± standard deviation. First row, PMCs vs. time; lineal fit. Second row, FG^+^RGCs vs. time; lineal fit. Third row, Brn3a^+^RGCs vs. time; lineal segmental fit. Fourth row, FG^+^RGCs vs. PMCs; lineal fit. *R* ^2^ is the correlation coefficient. *m* is the slope of the straight line. *X*_0_ is the day when the first linear phase of RGC loss changes to a second, slower one. *m*_1_ and *m*_2_ are the slopes of the first and second linear phases, respectively. Fifty percent appearance, clearance, or loss is the time (days) when, according to the mathematical analysis, half of the PMCs have appeared, half of the RGCs have been cleared from the tissue, or half of the RGCs have died, respectively. Left column ONC, right column ONT. **d** Quantitative topography. Line graphs where the mean number or percent of cells (*Y*-axis) counted after ONC (left column) or ONT (right columns) are plotted against the length of the retina (*X*-axis) being 0 the optic nerve and 6 mm the periphery. First row, number of Brn3a^+^RGCs. Second row, Brn3a^+^RGCs as percent of intact retinas (blue line). Third row, number of MCs, which are surveying MCs for intact retinas and PMCs for injured ones. Fourth row, percent of PMCs, being 100% the number of SMCs in intact retinas (blue line)

After ONC or ONT, traced RGCs disappear and PMCs appear (Fig. 2a–j). The morphology of MCs after the lesion changes from ramified to fusiform or ameboid. Fusiform or rod-like MCs are preferentially found in chains, along the RGC axons. Ameboid MCs are found more often engulfing a traced RGC which, normally, does not express Brn3a anymore (Figs. 2 and 3a).

As previously reported [2, 34], RGC loss after ONC is slower than after ONT, and this is observed with both identification methods (Table 2 and Fig. 3b, c). However, there are differences between the course of RGC death (Brn3a) and RGC clearance (FG).

**Table 2:** Injured retinas

		Retinal nerve fiber layer/ganglion cell layer					Inner plexiform layer			Ganglion cell complex (GCL + IPL)
		Brn3a^+^ RGCs	FG^+^ RGCs	PMCs	Non-PMCs	Total MCs	PMCs	Non-PMCs	Total MCs	
ONC	2 days (*n* = 8)	75,987 ± *4210*	80,459 ± *1552*	11 ± *5*						
	5 days (*n* = 8)	42,656* ± *5111*	67,302* ± *5901*	3116* ± *740*						
	9 days (*n* = 8)	25,105* ± *5789*	30,896* ± *1719*	13,401* ± *2549*						
	14 days (*n* = 12)	10,639* ± *3150*	15,755* ± *3359*	22,404* ± *1863*	2801 ± *350*	**23,206** ***± 1070***	760* ± *132*	4501 ± *546*	5261* ***± 404***	**28,467** ± ***1555***
	2 months (*n* = 4)	5457 ± *1724*	8580* ± *385*	10,497* ± *289*	2375 ± *408*	12,871* ***± 374***	2343 ± *304*	4694 ± *499*	7038* ***± 660***	19,910* ***± 948***
ONT	2 days (*n* = 8)	67,466*† ± *5539*	79,997 ± *1345*	43 ± *8*						
	5 days (*n* = 8)	31,938*† ± *4829*	54,290*† ± *4136*	5025* ± *1058*						
	9 days (*n* = 8)	18,015* ± *4542*	22,002*† ± *1336*	19,842*† ± *2534*						
	14 days (*n* = 12)	5515* ± *1101*	8627*† ± *1433*	22,092† ± *3514*	2258 ± *412*	***24,351***† ***± 2193***	941* ± *211*	4760 ± *801*	5702* ***± 792***	**30,053** ***± 2202***
	2 months (*n* = 4)	2708 ± *813*	2983* ± *232*	10,865* ± *584*	3280 ± *685*	14,145* ***± 410***	3971† ± *125*	3756 ± *356*	7728* ***± 332***	21,873* **±** ***415***

Mean number ± *standard deviation* of Brn3a^+^RGCs, FG^+^RGCs, phagocytic microglial cells (PMCs), and non-phagocytic microglial cells (non-PMCs, surveying and/or activated microglial cells that are not transcellularly labeled) in the same retinas within each group and time point. Total MCs is the sum of PMCs and non-PMCs

*MC* microglial cell, *FG* fluorogold, *RGCs* retinal ganglion cells, *ONT* optic nerve transection, *ONC* optic nerve crush, *RNFL/GCL* retinal nerve fiber layer + ganglion cell layer, *IPL/INL* inner plexiform layer + inner limit inner nuclear layer, *GCC* ganglion cell complex (RFNL/GCL + IPL/INL)

*Significant difference compared to previous time point (2 days or IPL 14 days vs. intact or tracing control retinas, Table 1)

†Significant difference between ONC and ONT at the same time point. One-way ANOVA and Tukey’s post hoc test, *p* < 0.05. The number of analyzed retinas to quantify GCL cells is shown in the second left column. For the IPL analysis, four retinas per group/time point were used

In bold font is shown the total number ± *SD* of MCs (PMCs plus non-PMCs) in the different retinal layers

Within each lesion, RGC loss is detected earlier with Brn3a than with FG. Indeed, half of the RGCs have died 5.6 days after ONC or 4.2 days after ONT, while half of the RGC clearance occurs 3 days later for both lesions (at 7.2 or 8.4 days respectively). Furthermore, RGC death adjusts to a segmental lineal regression with two phases of loss, one quick that lasts up to 7 days for ONC (daily loss of 7326 RGCs, *m*_1_) or to 5.8 days for ONT (*m*_1_, 9762 RGCs/day) and a slower one thereafter with a comparable rate of loss for both lesions (m2 values). For its part, RGC clearance is linear and the daily disappearance rate is similar between lesions (there are no significant differences in the slopes between ONC and ONT *p* = 0.59), albeit delayed for 1 day (see above) and less pronounced after ONC (5249 RGCs/day) than after ONT (5795 RGCs/day).

### PMC dynamics

PMC appearance highly correlates with FG clearance (Fig. 3c), but not as much with RGC death (Brn3a^+^RGCs vs. PMCs, regression coefficient 0.84, not shown). Incidentally, the FG^+^RGCs vs. PMCs correlation tells us that for each 3288 or 3044 cleared RGCs, there is an increase of 1000 PMCs, meaning that one PMC is able to engulf ~ 3 RGCs. This ratio can also be estimated from the slope of FG^+^RGCs vs. time by the slope of PMCs vs. time (i.e., 5249/1789 = 2.9).

**Fig. 4:**
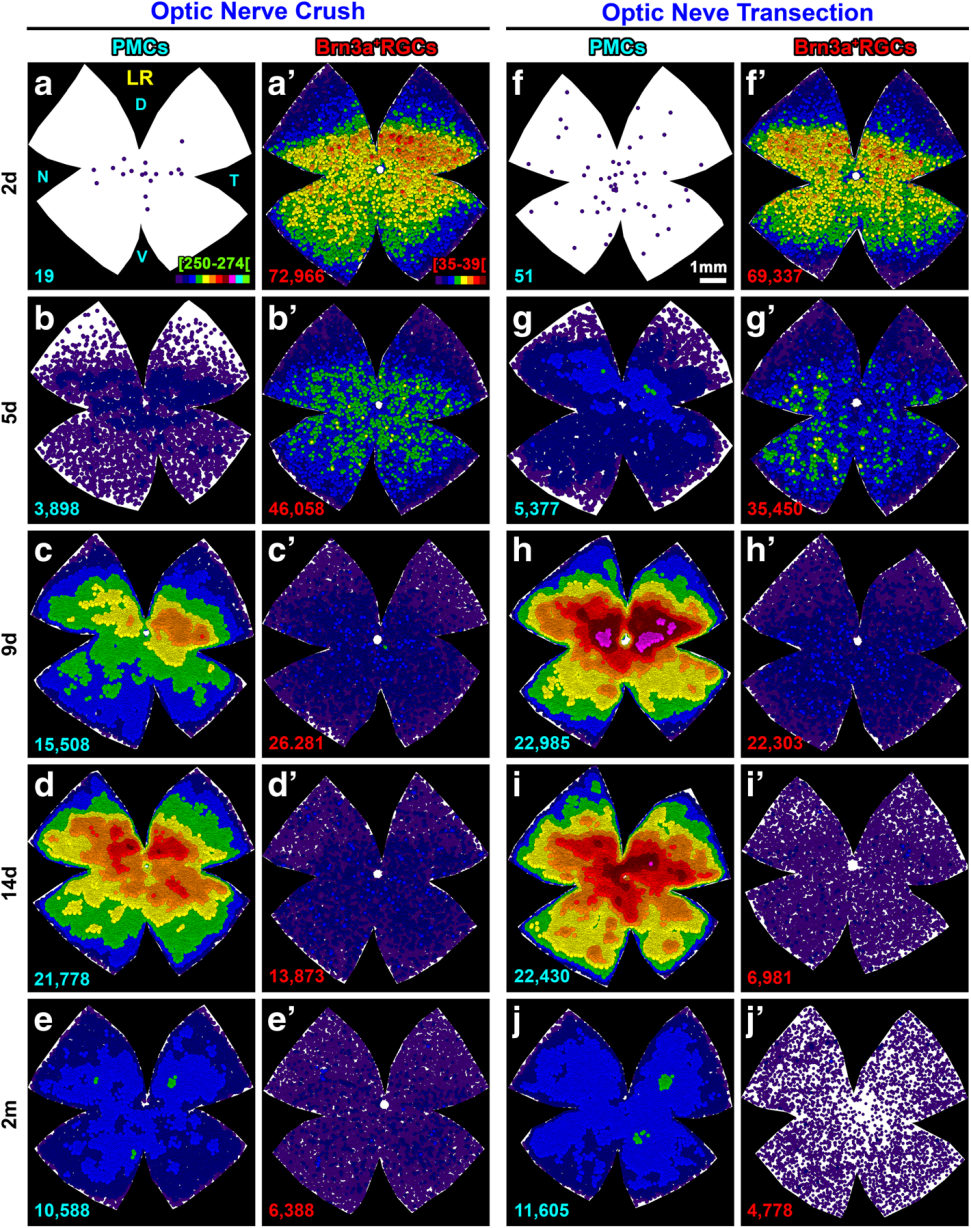
Topographic correlation of PMC appearance and RGC death. Neighbor maps showing the spatial distribution of PMCs (**a**–**j**) and Brn3a^+^RGCs (**a’**–**j’**) in the same retinas within each group and time point after optic nerve transection (**a**–**e’**) or crush (**f**–**j’**). Map description is in Fig. 1 legend

Quantitative analyses of cell topography show that more RGCs die in the central retina (Fig. 3d, first graph). Proportionately, though, their death is synchronous along the retina because the percent of loss is similar at any given distance from the optic nerve (Fig. 3d, second graph). In agreement, PMCs appear across the GCL, increasing in number with increasing post-lesion intervals, following the topographic pattern of RGC loss (Fig. 3d, compare the third graph with the first one). However, proportionally, the central retina bears a higher percent of PMCs than the periphery (Fig. 3d, fourth graph). This can be explained because there are more PMCs where more RGCs are dying, and this is clearly observed when the data are graphed as neighbor maps (Fig. 4); while RGC loss is diffuse, the emergence of PMCs seems centripetal being their density higher in the retinal areas where RGCs are more abundant (RGC distribution in an intact retina, Fig. 1g).

### Microglial migration between layers

**Fig. 5:**
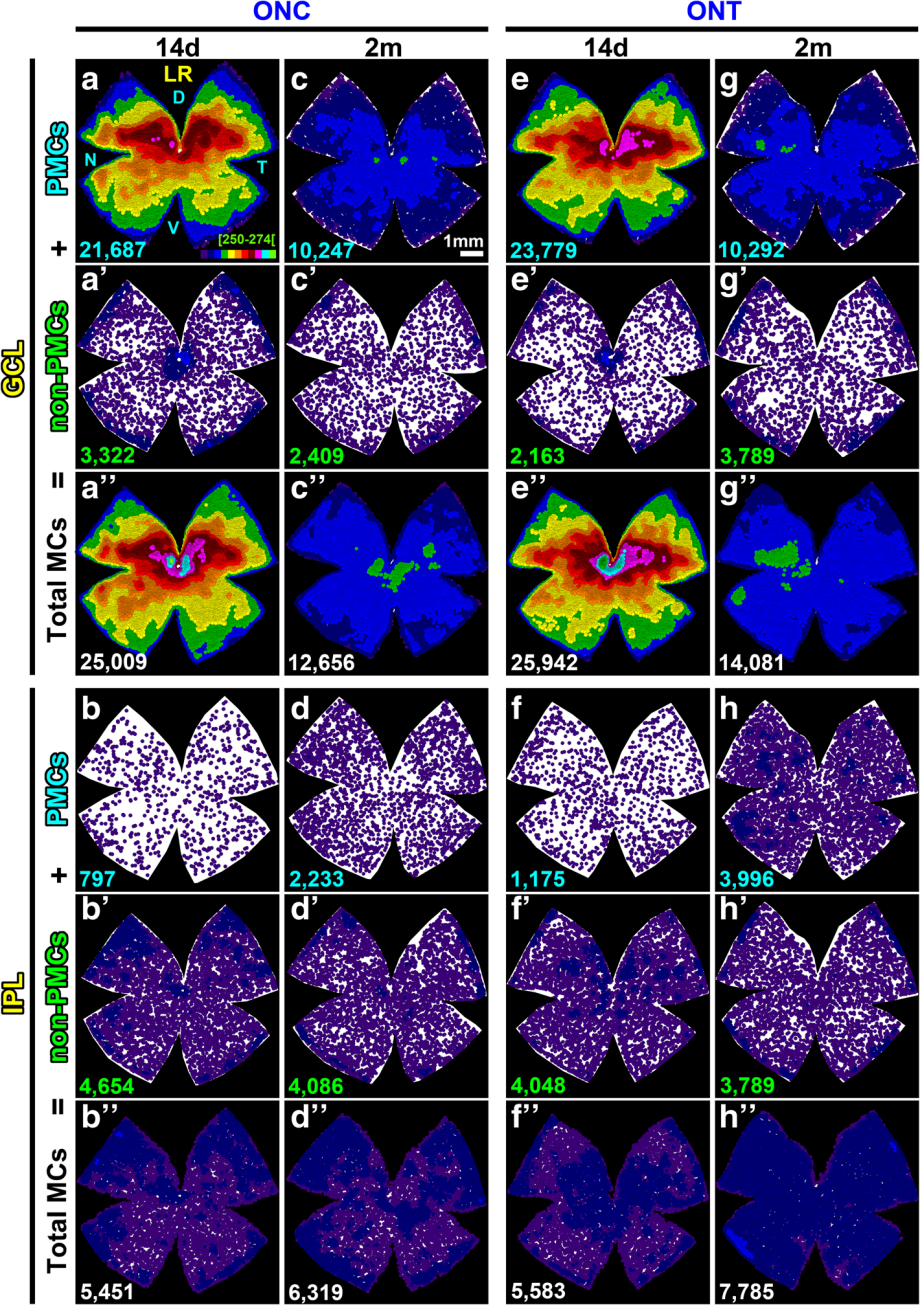
Migration of microglial cells between the ganglion cell layer and the inner plexiform layer after axotomy. Neighbor maps showing the spatial distribution of PMCs, non-PMC (non-phagocytic), or total MCs (sum of both) in the ganglion cell complex at 14 days and 2 months post-ONC (**a**–**d”**) or ONT (**e**–**h”**). Each column is the same retina, where MCs in the GCL and in the IPL have been analyzed. Map description is in Fig. 1 legend

PMCs peak at 14 days (Table 2). At this time point, ~ 90% of MCs in the GCL are PMCs which, as abovementioned, are more abundant in the areas of higher RGC density and death (Fig. 5a, e). The remaining 10% are non-PMCs and are distributed fairly homogenously, except for a dense accumulation around the optic nerve and along the extreme periphery (Fig. 5a’, e’).

**Fig. 6:**
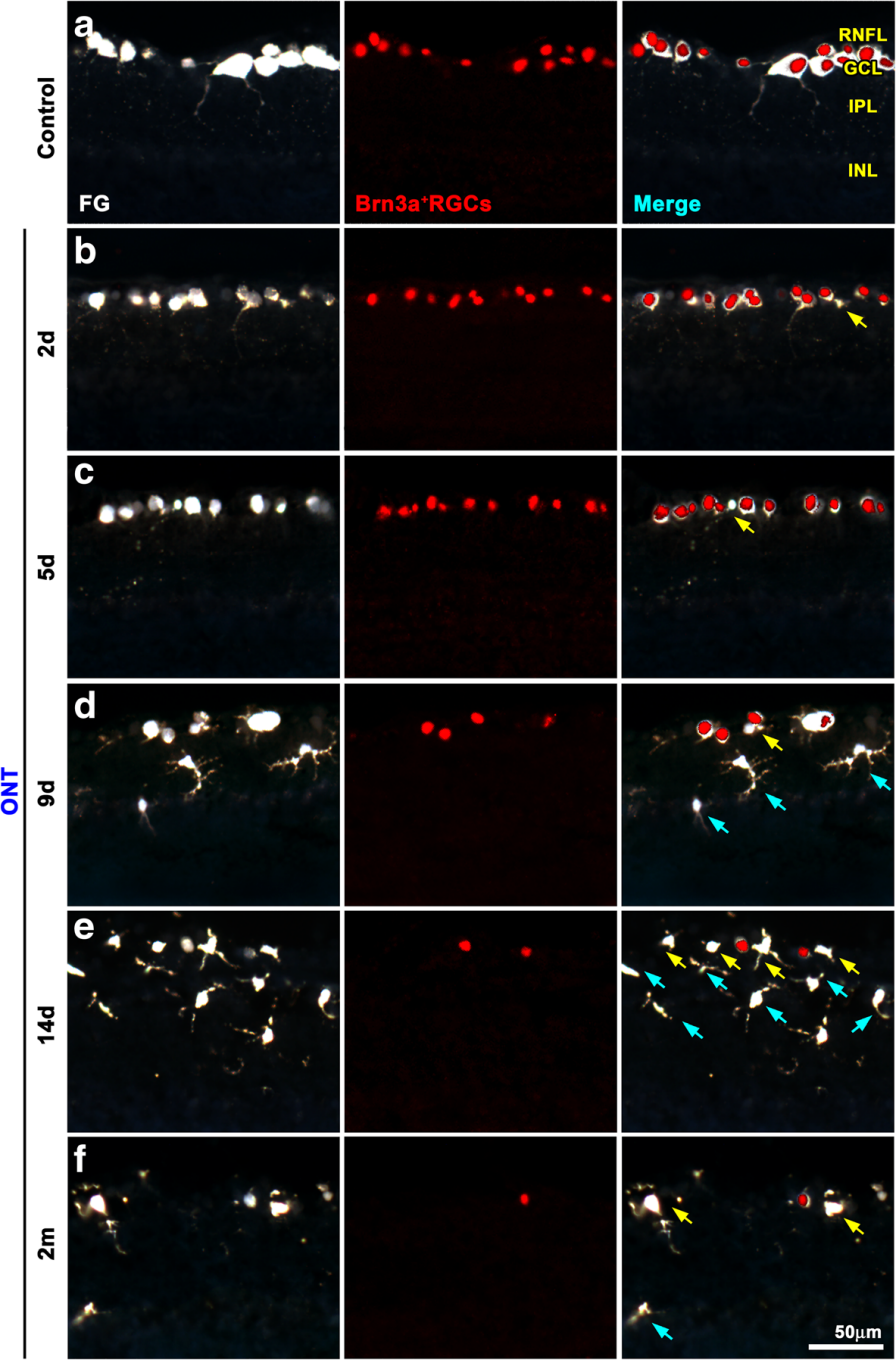
PMCs appear first in the ganglion cell layer and afterwards in the inner plexiform layer. Cross sections from control and injured traced retinas immunodetected with Brn3a. **a** Control. **b**–**f** Injured at increasing times after axotomy. PMCs are observed at 2 and 5 days in the GCL, and they become apparent in the IPL/INL from 9 days onwards. Yellow arrows point to PMCs in the ganglion cell layer and blue ones to PMCs in the plexiform and inner nuclear layers

At its highest, the number of PMCs + non-PMCs in the GCL surpasses the number of SMCs found in intact retinas by ~ 10,000. In parallel, there is a uniform decrease of MCs in the IPL (around 50% of their initial population) that justifies half of the excess observed in the GCL, suggesting that there is microglial migration between these layers (Table 2, Fig. 5b–b”, f–f”). Indeed, PMCs are observed orthogonally placed in the IPL and INL from 9 days onwards (Fig. 6).

Two months after the lesions, when RGC death has slowed down, the number of MCs in the GCL has decreased and is numerically (Table 2) and topographically (Fig. 5c–c”, g–g”) normal, albeit most of these MCs are still transcellularly labeled. For its part, the IPL has partially recovered by the addition of PMCs which, most probably, come from the GCL (Table 2, Fig. 5d–d”, h–h”). However, the IPL, and therefore the GC complex, are deficient on 3000–5000 MCs compared to that on intact retinas.

## Discussion

We describe here for the first time the numerical, mathematical, and topographical relationship between RGCs and MCs in healthy and injured rat retinas. The strengths of this work lie firstly on the fact that RGCs are the only retinal neurons that die after optic nerve axotomy [2, 43–45] and, secondly, on the analysis of whole populations in the same retinas, using markers of RGC viability and clearance in combination with the identification of phagocytic and non-phagocytic microglial cells. We have limited our study to the retinal layers where the RGC axons, somata, and processes are and focused the analysis on the phagocytic response rather than on microglial activation [46–49].

### Intact retinas

In agreement with previous reports, MCs are homogenously distributed in the GCL and IPL of healthy retinas [46, 49]. This topography reflects their surveying function, explained by the continuous mobility of MCs and their multidirectional projections used to maintain the distance between surrounding cells [50].

In traced but otherwise intact rat retinas, the number of PMCs depends on the time of tracing, as it has been shown in mice [21]. In these retinas, we did not observe a significant loss of Brn3a^+^RGCs (at least up to 2 months). Thus, the presence of PMCs can be explained by phagocytosis of the FG excreted by the traced RGCs, by pruning of RGC neurites, and/or by direct toxicity of the tracer on a few RGCs. Our study does not allow to discriminate among these reasons, but given the very low number of PMCs quantified in these control retinas, tracing itself does not influence PMC quantification after injury.

### Contralateral uninjured retinas

In mice, after unilateral ON axotomy, there is a strong PMC reaction in the contralateral (uninjured) retina that is constant, scattered, and independent of the rate of RGC survival in the axotomized retina [21, 24]. In rats, a contralateral response involving activation of MCs, astrocytes, and Müller cells has been observed after axotomy [47, 51–54], or ocular hypertension [55], but to our knowledge, this is the first report describing that in this species, there is not a PMC reaction. At present, we do not know why, but it is possible that the scarce retino-retinal projection is implicated because in adult rats, this projection is missing in some animals and, when present, is smaller than in mice [56–58]. Thus, retino-retinal RGCs, which are directly affected by the axotomy, could transmit a danger signal that would spread along the retina, recruiting and activating MCs. These signals would not be sufficient to elicit a PMC response in those rats with very few retino-retinal RGCs, and inexistent in those animals without them. In addition, these signals might combine with a systemic inflammatory response that would account for the inflammatory reaction observed in both species.

### Microglial morphology

The role of MCs in neurodegenerative diseases is controversial, but it is clear that they are not simple bystanders [27]. For one part, MCs restore tissue homeostasis, keeping the neuronal interactions as healthy as possible [9, 10], and their suppression improves axonal regeneration and RGC survival after axotomy [59]. On the other hand, MC over-activation may be damaging [31], as they act as mediators of the inflammatory response observed in several retinal degenerative diseases (reviewed in [60]), although in this respect there is some debate [61].

Upon neuronal death, MCs change their morphology from a surveying ramified shape to an ameboid-like one, M1 phenotype, which is considered harmful [28, 62–65]. Here, we show that ameboid PMCs are often found engulfing RGC somas. We also found phagocytic rod-shaped MCs, especially along the intraretinal axons, in agreement with other works describing that rod-shaped MCs are induced by the interruption of axonal transport after insults such as axotomy or ocular hypertension [46, 66], but also during aging [67]. Rod-shaped MCs bear the M2 phenotype, and although their function is unknown, it is hypothesized that they protect the degenerating neurons [29, 67, 68]. In apparent contradiction with that hypothesis, we show here that rod-shaped MCs are or have been involved in the phagocytic response. In other words, either they engulf FG in their rod-shape state, or they could change from an ameboid phagocytic state to a protective rod-shape during the course of RGC degeneration. Because FG is accumulated in the phagolysosomes of MCs for a long time after cleansing the tissue, at least 2 months as shown here, we cannot rule out either possibility.

### RGC death and clearance

One of the most interesting results of this work is the 3 days delay between RGC death and RGC clearance that was observed after both lesions. This means that independently of the course of RGC loss (slower after ONC than after ONT), 3 days are needed for the MCs to become phagocytic. Therefore, to assess RGC death, tracing is not the best approach, particularly when manipulating MCs as neuroprotective tool, because MC inhibition may further delay the removal of dead RGCs from the tissue.

### PMC migration

At 14 days, the number of PMCs in the GCL exceeds the population of SMCs in intact retinas by > 10,000. There are several theories to explain this increase: (I) microglial proliferation [69–71], (II) extravasation of monocytes [72], and (III) migration from other retinal layers [59]. With our data, we cannot rule out (I) or (II). We found at 14 days, non-PMCs spread along the GCL, except around the optic nerve and the retinal periphery where they accumulated. They may reflect either local division, or retinal infiltration of monocytes from the ciliary bodies [73], and the peripapillary region. It is worth noting that these non-PMCs may or may not be activated, and although they have not engulfed RGC somas, they may have phagocytosed RGC axons, which do not accumulate FG. However, we do demonstrate that there is MC migration between the IPL and the GCL that would account for half of the MC surplus. Interestingly, 2 months after either lesion, the number of MCs in the GCL decreases, increasing in the IPL with cells from the GCL as the returning ones are PMCs. Finally, while the number of MCs in the GCL is back to normal, the IPL is short of ~ 4000 MCs. Have the missing MCs died? Or have they left the retina? We think that the second possibility is more plausible, because MC death would probably cause a second wave of MC activation and phagocytosis.

### Topography

Our state-of-the-art topographical analyses show that more PMCs accumulate in the retinal regions where there is, proportionally, more RGC death. It is important to have in mind that RGC death is diffuse along the retina, meaning that RGCs die synchronously, but more of them die where there are more. Thus, there must be signals from the degenerating neurons and sensors in the MCs that inform of the amount of cell bodies to be removed, and upon them, more or less MCs migrate to the injured zone, having into account that one MC is able to engulf three RGC somatas. All in all, these results indicate that phagocytosis is activated by the death of RGCs rather than by the inflicted axonal damage.

Finally, our present data together with a previous work in mice [21] suggest that after optic nerve axotomy, MCs perform a cleansing role, but there is no indication of them being involved in the death of RGCs, at least during the quick phase of loss. In fact, Hilla et al. [74] have recently demonstrated, using an in vivo approach to deplete retinal MCs, that these cells do not play an active role on the death of RGCs after optic nerve axotomy.

## Conclusion

In conclusion, after axotomy, RGC death precedes RGC clearance and there is an inverse correlation between RGC loss and PMC appearance, both numerically and topographically. Finally, to cope with the massive axotomy-induced RGC loss, MCs travel from the IPL to the GCL. The system appears restored at 2 months, when the GCL presents a normal number of MCs and the IPL has remodeled to its new status.

## Abbreviations

ARVO: Association for Research in Vision and Ophthalmology
Brn3a: Brain-specific homeobox/POU domain protein 3a
DAPI: 4′,6-Diamidino-2-phenylindole
dRGC(s): Displaced retinal ganglion cells
FG: Fluorogold
GCL: Ganglion cell layer
Iba1: Ionized calcium-binding adapter molecule 1
INL: Inner nuclear layer
IPL: Inner plexiform layer
MCs: Microglial cells
*n*: Sample size
Non-PMC(s): Non-phagocytic microglial cells
OCT: Optimal cutting temperature
ON: Optic nerve
ONC: Optic nerve crush
ONL: Outer nuclear layer
ONT: Optic nerve transection
OPL: Outer plexiform layer
PMC(s): Phagocytic microglial cells
RGC(s): Retinal ganglion cells
RNFL: Retinal nerve fiber layer
SCi: Superior colliculi
SMC(s): Surveying microglial cells
vs.: Versus
